# Evaluating the association of COVID-19 restrictions on discharge planning and post-discharge outcomes in the community hospital and Singapore regional health system

**DOI:** 10.3389/frhs.2023.1147698

**Published:** 2023-09-05

**Authors:** Shermain Chia, Jiawen Xia, Yu Heng Kwan, Zhui Ying Lim, Chuen Seng Tan, Sher Guan Low, Bangyu Xu, Yu Xian Loo, Lai Yee Kong, Chee Wai Koh, Rachel Marie Towle, Su Fee Lim, Sungwon Yoon, Sharna Si Ying Seah, Lian Leng Low

**Affiliations:** ^1^ Post-Acute and Continuing Care, Outram Community Hospital, Singapore, Singapore; ^2^ Research and Translational Innovation Office, Singhealth Community Hospitals, Singapore, Singapore; ^3^ Health Services and Systems Research, Duke-NUS Medical School, Singapore, Singapore; ^4^ Department of Pharmacy, National University of Singapore, Singapore, Singapore; ^5^ Population Health & Integrated Care Office, Singapore General Hospital, Singapore, Singapore; ^6^ Saw Swee Hock School of Public Health, National University of Singapore, Singapore, Singapore; ^7^ Post-Acute and Continuing Care, Sengkang Community Hospital, Singapore, Singapore; ^8^ Medical Social Services, Sengkang Community Hospital, Singapore, Singapore; ^9^ Medical Social Services, Outram Community Hospital, Singapore, Singapore; ^10^ Specialty Nursing, Population Health & Integrated Care Office, Regional Health System, Singapore, Singapore; ^11^ Regional Health System Community Nursing, Population Health & Integrated Care Office, Singapore General Hospital, Singapore, Singapore; ^12^ Department of Family Medicine and Continuing Care, Singapore General Hospital, Singapore, Singapore; ^13^ Singhealth DukeNUS Family Medicine Academic Clinical Program, Regional Health System, Singapore, Singapore

**Keywords:** COVID-19, discharge planning, public health, care continuity, lockdown

## Abstract

**Objectives:**

The COVID-19 is a global health issue with widespread impact around the world, and many countries initiated lockdowns as part of their preventive measures. We aim to quantify the duration of delay in discharge to community from Community Hospitals, as well as quantify adverse patient outcomes post discharge pre and during lockdown period.

**Design and methods:**

We conducted a before-after study comparing the length of stay in Community Hospitals, unscheduled readmissions or Emergency Department attendance, patients' quality of life using EQ5D-5l, number and severity of falls, in patients admitted and discharged before and during lockdown period.

**Results:**

The average length of stay in the lockdown group (27.77 days) were significantly longer than that of the pre-lockdown group (23.76 days), *p* = 0.003. There were similar proportions of patients with self-reported falls post discharge between both groups. Patients in the pre-lockdown group had slightly better EQ-5D-5l Index score at 0.55, compared to the lockdown study group at 0.49. Half of the patients in both groups were referred to Community Care Services on discharge.

**Conclusion:**

Our study would help in developing a future systematic preparedness guideline and contingency plans in times of disease outbreak and other similar public health emergencies.

## Introduction

The novel Coronavirus disease 2019 (COVID-19) is caused by the Severe Acute Respiratory Syndrome Coronavirus 2 (SARS-CoV-2) (1), and has become a global health issue with widespread impact on countries around the world. In March 2020, the World Health Organization (WHO) declared it a pandemic due to its alarming rates of spread and severity (2). As of August 2022, there have been 587 million confirmed cases, and 6.4 million deaths reported globally (3). Many countries around the world initiated lockdowns as part of their preventive measures, with the aim to curb the spread of the virus by limiting people's movement.

Intermediate and long-term care (ILTC) services include a range of healthcare services outside of acute hospitals, typically required for patients who need further care after acute hospitalizations (4). They consist of home-based (such as home medical/nursing/palliative care and Meals-On-Wheels), centre-based (such as day care, day hospice), and residential services [such as community hospitals (CHs), nursing homes]. CHs in Singapore are intermediate care facilities, vital to the continuum of health services that are incorporated into the healthcare landscape to bridge acute and primary care. Similar to CHs in the United Kingdom (5), they are pivotal in delivering both health and social care to optimize patients' activities of daily living in preparation for community re-integration or long-term care stay (6). On discharge from CH, should transfer to or follow-up with long term care facilities such as nursing homes, senior care services and day rehabilitation centers be warranted, the CH team would apply via the Agency for Integrated Care portal after getting patients' and family agreement.

A search on the current literature reveals extensive research into the pathology, diagnosis, prevention and management of COVID-19 (7–9). We also found studies delving into the impact of COVID-19 on various at-risk groups of patients who were not infected with the virus, such as patients with dementia (10, 11), cancer (12), and eating disorders (13, 14). However, there was scant research investigating how lockdown measures have impacted discharge planning and continuity of integrated care for patients after being discharged from intermediate care facilities such as CHs during the lockdown period.

In an effort to help fill this gap, our study aims to (i) quantify the difference in duration of stay at the CHs between the pre-lockdown and the lockdown periods, as well as (ii) quantify the difference in adverse patient outcomes after discharge to community from CHs between the pre-lockdown and during lockdown periods.

## Materials and methods

### Design

This was a before-after study approved by the Institutional Review Board, and is part of a larger mixed-methods study (15).

### Setting and participants

CHs in Singapore provide rehabilitation care and post-acute care to patients recovering after an acute illness. Participants were recruited from two large CHs with a total of 848 beds, which are part of a Regional Health System in Singapore serving more than 50% of the country's population with its network of acute hospitals, national specialty centres, CHs and polyclinics. Typical patients in a CH would be deconditioned after a major illness, such as stroke, hip fracture, Parkinson's disease, heart failure, to name a few.

Despite efforts to limit the spread of COVID-19 through aggressive contact tracing, strict isolation methods and vigorous testing in Singapore, there was an increasing trend of community transmission with outbreaks in foreign worker dormitories (16). This led to the Singapore government to impose an elevated set of safe-distancing measures in the form of a country-wide lock down, termed “Circuit Breaker” in early April 2020 (17).

As part of the lockdown, services deemed to be “non-essential” were scaled down or halted. Residential and home-based community care services such as nursing homes, psychiatric rehabilitation homes, inpatient or home palliative care continued to function, while home personal care services were scaled down (18). Senior care centers, day rehabilitation centers, medical escort services and day hospices were closed temporarily (18). Public and private acute hospitals, CHs, polyclinics were deemed to be essential and remained open, though with tightened visitation policies (19). For example, nearly all visitors were prohibited to hospitals except in special circumstances such as for dangerously ill patients. The discharge process would understandably be delayed in view of disruption of training for caregivers and changes in therapy sessions.

We examined patients aged 21 years old (minimum age for patients to be admitted to CHs) and above who were admitted and then discharged in sub-acute and rehabilitation wards in the two CHs between 7 April 2020 to 31 July 2020 (study group during lockdown), a period during which Singapore was under lockdown measures. The outcome measures from this group of patients were compared with those admitted and then discharged from 1 Nov 2019 to 29 Feb 2020 (pre-lockdown period).

### Data collection

Baseline demographic characteristics during recruitment including age, gender, marital status, ethnic group etc were collected, as well as Charlson Comorbidity Index (CCI) Score, Admission Diagnosis Related Group (DRG), length of hospital stay (LOS) and details of referral to Community Care Services (CCS) through medical records review.

Follow-up data were obtained via phone calls at 3 timepoints (30 days, 90 days, 180 days post discharge) for lockdown group patients and at least 1 timepoint (90 days and if possible, 180 days post discharge) for the pre-lockdown group. Patients who were discharged to long-term care facilities such as nursing homes, had hearing difficulties or faced language barriers when communicating with our study team members were excluded from this study. Due to the time-sensitive nature of this study and time required for the research grant and ethics approval, data collection of self-reported outcomes at 30 days was not possible for some of the lockdown group participants. However, key outcome measures such as length of stay, Emergency Department (ED) attendance or unscheduled readmissions within 180 days of discharge were extracted via electronic health records.

### Outcome measures

The primary outcome measure was LOS (days) in the respective CHs. Secondary outcome measures were 180 days unscheduled readmissions or ED visits to an acute hospital, number and severity of falls (using the National Database of Nursing Quality Indicators® developed by the American Nurses Association (ANA-NDNQI) for fall related injuries categories (20)) at the most distal of the 3 timepoints post discharge available, and patient's quality of life [using EQ-5D-5l (21)] at 180 days post discharge.

EQ-5D-5l consist of 5 dimensions (mobility, self-care, usual activities, pain/discomfort, anxiety/depression), each with 5 levels (no problems, slight problems, moderate problems, severe problems and extreme problems), which can be converted to an Index score that reflects one's health status and quality of life. A higher index score corresponds to a better quality of life and better health. EQ-5D-5l VAS is a 0—100 scale where patients are asked to indicate their overall health on the day of questionnaire completion, with 100 as the best possible health imaginable (22). Incorporating both EQ-5D-5l Index score and VAS as outcomes measures in our study gives a more holistic picture of participants' quality of health.

We defined unscheduled readmissions or ED visits as any admissions or visits due to an unforeseen or non-elective cause, within 180 days of discharge from index community hospital admission.

### Data analysis

Categorical variables were summarized using counts and percentages, while continuous variables were summarized using means and standard deviations. To assess the association of group status (pre-lockdown vs. lockdown) with categorical and continuous variables, bivariate analyses were performed by using the Chi-square test and two-sample *t*-test respectively. Linear regression models were performed to assess the association between group status and length of stay in days. A simple linear regression model with only group status was first considered and provided an unadjusted analysis. Subsequently, a multiple linear regression model was considered that also included significant variables in the bivariate analyses such as gender and CCI as predictors to provide an adjusted analysis. The analyses were conducted using R software, Version 4.1.3, with *p*-value < 0.05 was considered as statistically significant.

## Results

### Participants

**Figure 1:**
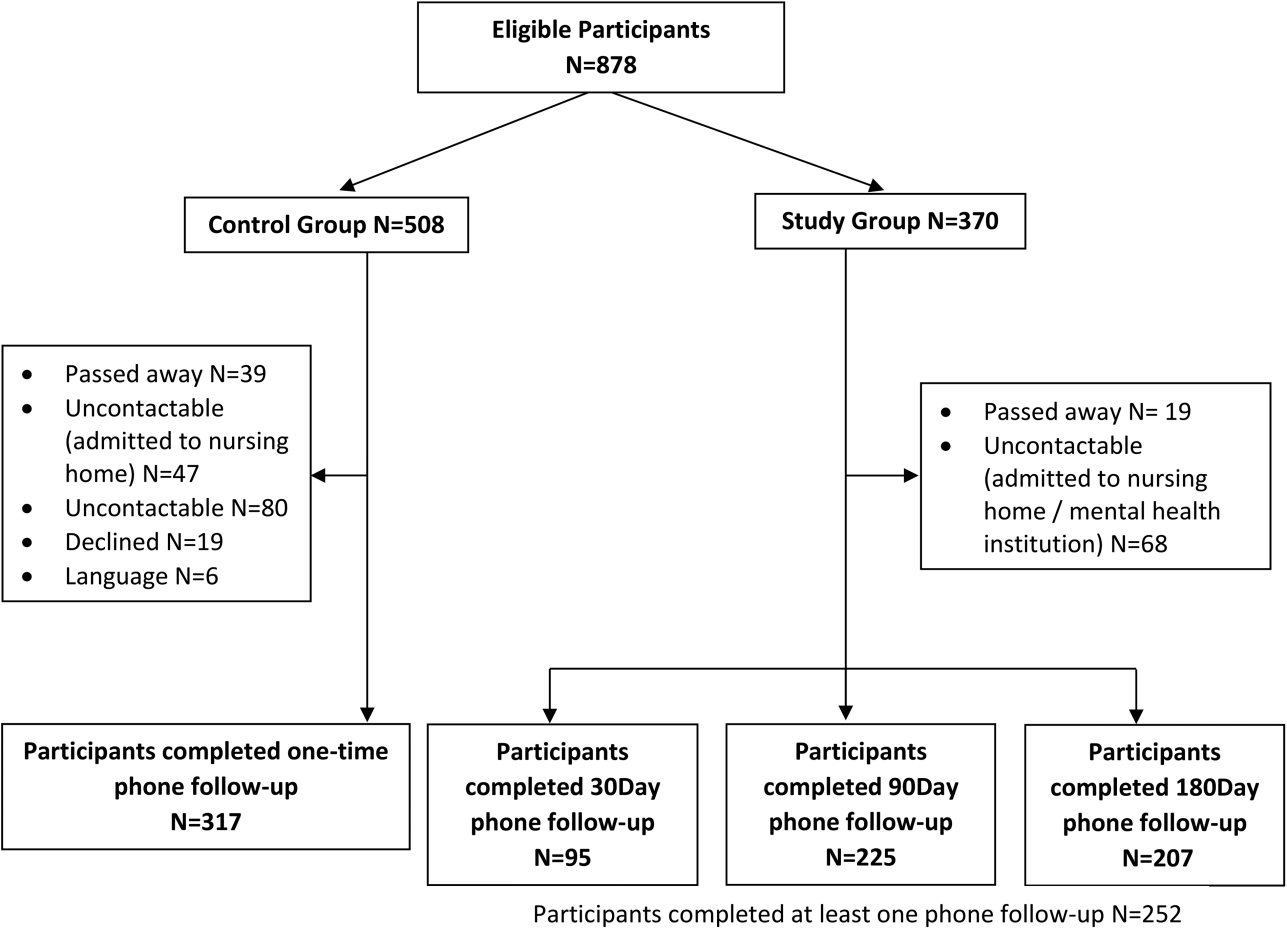
Participant flowchart.

A total of 878 patients fulfilling criteria were admitted and discharged during the study period, with 508 from the pre-lockdown group and 370 from the lockdown group (Figure 1). Amongst these, 191 patients (37.6%) from the pre-lockdown group and 77 patients (20.8%) from the lockdown group were lost to follow-up for patient-reported outcomes at all three time points due to reasons such as being uncontactable, declined follow-up phone calls, language barriers or death. Overall, 317 patients from the pre-lockdown group completed one-time phone follow-up. In the lockdown group, 95 patients completed 30 days phone follow-up, 225 patients completed 90 days phone follow-up, and 207 patients completed 180 days phone follow-up.

The baseline characteristics of the study population are shown in Table 1. There were more female patients in both groups, at 312 (61.4%) for the pre-lockdown group, and 198 (53.5%) for the lockdown group. The mean age of both groups was the same at 75 years old. It is notable that patients in the lockdown group had a statistically significant higher CCI Score (*p* < 0.001). We have also included the common comorbidities seen among patients in the pre-lockdown and lockdown group in Table 2.

**Table 1:** Patient characteristics.

Patient characteristics *n* = 878	Control *n* = 508	Study *n* = 370	*P*-value
Gender, *n* (%)			
Male	196 (38.6)	172 (46.5)	.01909
Female	312 (61.4)	198 (53.5)	
Age group, *n* (%)			
65 and above	417 (82.1)	305 (82.4)	.8947
Below 65	91 (17.9)	65 (17.6)	
Age in years, mean (SD^a^)	75.14 (11.18)	75.14 (12.33)	.9997
Ethnic group, *n* (%)			
Chinese	433 (85.2)	305 (82.4)	.2625
Non-Chinese	75 (14.8)	65 (17.6)	
Marital status, *n* (%)			
Single	85 (5.7)	68 (18.4)	.2343
Married	141 (27.8)	84 (22.7)	
Unknown	282 (55.5)	218 (58.9)	
LOS^b^ in days, mean (SD)	23.76 (16.09)	27.77 (17.73)	0.003 (Adjusted)
CCI^c^ score, mean (SD)	4.95 (2.64)	6.06 (2.82)	<.001
No. of unscheduled readmissions/ED visits (180 day), mean (SD)	1.54 (0.89)	1.55 (1.05)	.925

^a^ SD, Standard deviation.

^b^ LOS, Length of hospital stay.

^c^ CCI, Charlson Comorbidity Index.

**Table 2:** Common comorbidities among patients in control and study group.

Common comorbidities among patients, *n* (%)	Control *n* = 317	Study *n* = 252	*P*-value
Myocardial infarction	40 (12.62)	39 (15.48)	0.3913
Congestive heart failure	27 (8.52)	20 (7.94)	0.923
Cerebrovascular disease	57 (17.98)	78 (30.95)	0.0004
Dementia	31 (9.78)	39 (15.48)	0.054
Diabetes mellitus	99 (31.23)	98 (38.89)	0.069
Chronic kidney disease	45 (14.2)	50 (19.84)	0.0929

### Primary and secondary outcomes

The average LOS in the lockdown study group (27.77 ± 17.73 days) were significantly longer than that of the pre-lockdown group (23.76 ± 16.09 days), at *p* = 0.003 (Table 1). The linear regression model was adjusted for gender and CCI Score as shown in Table 3. Patients admitted during COVID-19 were more likely to stay 3.5 more days than patients who were admitted during pre-COVID-19 period.

**Table 3:** Association between COVID-19 study group status and length of stay.

	Coefficient	95% Confidence interval	*P*-value
COVID-19^a^	3.4873	1.154–5.820	0.003

^a^ The linear regression model was adjusted for gender and CCI score due to the statistical significance at baseline between COVID-19 study group and control group.

There was a similar proportion of patients with self-reported falls post discharge at 180 days between both groups at around 18%, with an average of 0.3 falls per participant (Table 4). In terms of quality of life, patients in the pre-lockdown group had slightly better EQ-5D-5l Index score at 0.55 ± 0.44, compared to the lockdown study group at 0.49 ± 0.49 (Table 4), although there is no statistical significance. In addition, pre-lockdown group patients had significantly better EQ-5D-5l VAS score (68.03 ± 18.79) than lockdown study group patients (57.17 ± 30.20), *p* < 0.01.

**Table 4:** Self-reported falls and quality of life based on 569 patients surveyed in this study.

	Control *n* = 317	Study *n* = 252	*P*-value
Self-reported falls			
No. of self-reported falls post-discharge, *n* (%)	59 (18.61)	46 (18.25)	0.9129
No. of falls per participant, mean (SD^a^)	0.33 (0.78)	0.3 (0.95)	0.657
Quality of life based			
EQ-5D-5l index score, mean (SD^a^)	0.55 (0.44) (2 with no values)	0.49 (0.49) (48 with no values)	0.1289
EQ-5D-5l VAS score, mean (SD^a^)	68.03 (18.79) (33 with no values)	57.17 (30.20) (36 with no values)	<0.001

^a^ SD, standard deviation.

As can be seen from Table 5, approximately half of the patients in both the pre-lockdown and lockdown group were referred to Community Care Services on discharge, with the majority being centre-based care such as day care or day rehabilitation. Nearly two-thirds of patients from both groups require caregivers, of which half were foreign domestic workers.

**Table 5:** Discharge destination, community care services and caregiver needs.

	Control *n* = 317	Study *n* = 252
Patients discharged home with no community care services, *n* (%)	153 (48.26)	130 (51.59)
Patients referred to community care services, *n* (%)	164 (51.74)	122 (48.41)
Community care services referred per person, mean (SD^a^)	0.55 (0.57)	0.55 (0.62)
Type of community care services referral	(175)	(149)
Centre based care (includes day care and day rehabilitation)	134	87
Home based care (includes home medical, home nursing, home palliative care, home personal care, home therapy)	17	38
IHDC^b^, ICS^c^	14	12
Meals on wheels and medical escort	9	10
Residential care (short stay unit)	1	2
Community care services rendered post-discharge, *n* (%)	104 (63.41)	85 (69.67)
Require caregiver, *n* (%)	195 (61.51)	162 (64.29)
Require new caregiver post-discharge, *n* (%)	78 (24.61)	38 (15.08)
Caregiver relationship		
Spouse	37 (18.97)	27 (16.67)
Child/child-in-law	28 (14.36)	39 (24.07)
Parent/grandchildren	0	3 (1.85)
Foreign domestic worker	102 (52.31)	75 (46.30)
Others (siblings, relatives, friends etc.)	28 (14.36)	17 (10.49)

^a^ SD, Standard deviation.

^b^ IHDC, Integrated Home and day care.

^c^ ICS, Interim care services.

## Discussion

In our study, we found that patients staying in CHs during the lockdown period had statistically significantly longer LOS in the CHs than those during the pre-lockdown period. Limitations to access in follow-up care like day rehabilitation centres and medical escort services during lockdown may likely have prolonged the discharge process and impacted on continuity of integrated care. Other postulations to explain the longer LOS observed include delay due to lockdown restrictions such as new foreign domestic workers not being able to enter the country. The impact of COVID-19 pandemic measures and the subsequent disruption of community care services on discharge planning and continuity of integrated care on various stakeholders such healthcare workers, community partners and patients' caregivers, was explored in the qualitative component of our study through semi-structured interviews (15). Various studies have shown that higher CCI have been associated with a longer length of stay (23–25), and our linear regression model was adjusted for CCI due to statistical significance at baseline between pre-lockdown group and lockdown group.

Additionally, our study revealed that the patients staying in CHs during the lockdown period had a higher CCI Scores than those staying there during the pre-lockdown period. This suggests that more medically complex patients were admitted and discharged to CHs during the lockdown period. One possible explanation for this would be that acute hospital physicians tried to discharge patients back home as far as possible to reduce the risk of transmission in a facility. Hence, patients who were admitted to the CHs were more medically complex and also unable to be discharged directly to home from acute care. It is also possible that patients with mild illnesses avoided admission to CH for fear of COVID-19 transmission. This is alluded to in an Italy study whereby authors found a dramatic decrease in emergency department access during COVID-19 period, especially after lockdown, with the reduction mostly from patients who decided to seek emergency department care directly (26). This was attributed at least partly to fear of being infected with COVID-19. Other studies have also showed similar findings (27, 28).

In an effort to circumvent the safe distancing measures and to facilitate communication for better continuity of care, telemedicine had gained popularity due to the reduction in risk of COVID-19 transmission and the Singapore government's Smart Nation initiative (29). At MyDoc, a telemedicine platform headquartered in Singapore, the number of daily active users rose 60% in February and more than doubled again in March 2020 (30). However, its uptake was found to be limited by a number of factors, including patient-related barriers such as limited digital literacy among older adults, hearing impairments and inability to adequately describe care needs and symptoms (15, 31). Measures to educate the less tech-savvy population as well as to promote access to such telemedicine services could help improve utilisation of telemedicine, which have been demonstrated in studies to help promote disease management and self-care in older patients (32, 33). This would be exceptionally useful in the face of the various safe distancing measures (34).

Furthermore, our study found lower EQ-5D-5l Index score and EQ-5D-5l VAS score for patients in the lockdown group, suggesting a lower quality of life in this group of patients. The effect of the pandemic on quality of life was further explored in several studies (35–37), and reported the far-reaching effects of COVID-19 beyond just healthcare-related outcomes.

As part of the efforts to improve its pandemic preparedness against future infectious disease outbreaks, the Singapore Ministry of Health established PREPARE, or the Programme for Research in Epidemic Preparedness and Response (38). This workgroup is tasked with developing a national epidemic Research and Development plan that helps generate methods and tools to respond to future infectious disease outbreaks. Our study has shown a possible association of longer length of stay in CH and worse quality of life among patients in the lockdown group while they were under COVID-19 restrictions. This could spur on efforts by the PREPARE workgroup to design measures that identify and help mitigate such effects.

To our knowledge, this is one of the first studies that attempts to quantify the duration of delay in discharge as well as adverse discharge outcomes of patients from CHs before and after COVID-19 lockdown period. However, the results of our study should be interpreted in the context of its limitations. In view of the retrospective nature of the study, it is susceptible to recall bias among the pre-lockdown group participants and their caregivers due to time lag between study and patients’ discharge date. We attempted to minimize this by using objective measures such as LOS and 180 days unscheduled readmissions or ED attendance back to an acute hospital. These were obtained via electronic health records and hence were not subjected to recall bias.

Additionally, despite the best of our efforts, we noted a significant proportion of patients lost to follow up (37.5% in the pre-lockdown group and in the 23.5% lockdown group). This could possibly influence the findings. Future studies are needed to further assess the long-term impact of lockdown measures.

## Conclusion

Pandemics such as COVID-19 had posed challenges to governments and its healthcare systems around the world. Our study findings quantified the impact of COVID-19 and safe distancing measures had on discharge planning in CHs and patient outcomes. The importance of continuity of integrated care services for patients cannot be overly emphasized, and we believe findings from our study would inform the future development of systematic emergency preparedness guidelines and contingency plans in times of disease outbreak and other similar public health emergencies. This would help to minimize disruption of integrated care throughout the different levels and sites of care within the health system. Exploring the feasibility and barriers to adopting innovative models of care for different patient segments will also help to build a more inclusive society.

## Data Availability

The raw data supporting the conclusions of this article will be made available by the authors, without undue reservation.
